# A new method for discovering EMAST sequences in animal models of cancer

**DOI:** 10.1038/s41598-018-32057-2

**Published:** 2018-09-13

**Authors:** Nitya Bhaskaran, Jennifer Luu, Scott T. Kelley, Mohammad W. Khan, Priyadarshini Mamindla, Kathleen L. McGuire

**Affiliations:** Department of Biology, Molecular Biology Institute, San Diego State University, San Diego, CA 92182 USA

## Abstract

Elevated Microsatellite Alterations at Selected Tetranucleotide repeats (EMAST) occur in up to 60% of colorectal cancers and may associate with aggressive and advanced disease in patients. Although EMAST occurs in many cancer types, current understanding is limited due to the lack of an animal model. Reported here is the design and implementation of an algorithm for detecting EMAST repeats in mice. This algorithm incorporates properties of known human EMAST sequences to identify repeat sequences in animal genomes and was able to identify EMAST-like sequences in the mouse. Seven of the identified repeats were analyzed further in a colon cancer mouse model and six of the seven displayed EMAST instability characteristic of that seen in human colorectal cancers. In conclusion, the algorithm developed successfully identified EMAST repeats in an animal genome and, for the first time, EMAST has been shown to occur in a mouse model of colon cancer.

## Introduction

A specific form of microsatellite instability (MSI), termed EMAST, has been studied in cancer for the last several years (for review see^1–3^). It is most frequently observed in colorectal cancer (CRC) but has been found in other cancer types as well, including breast, pancreatic, bladder, and non-small cell lung cancer. EMAST is characterized by the insertion or deletion of di-, tri-, and tetranucleotide repeats in repetitive coding and non-coding DNA. EMAST has been observed in up to 60% of sporadic CRC cases^3–7^. Some studies have suggested that EMAST^+^ tumors are associated with advanced staging at diagnosis, increased size, and/or higher rates of cancer recurrence^3,5,8–10^. MSI has been identified as an independent marker of patient prognosis in CRC and EMAST has also been found to associate with poor patient outcome in some studies^1,9^. EMAST^+^ tumors overlap with MSI-High tumors, which result from DNA mismatch repair (MMR) defects due to the importance of this repair mechanism in maintaining microsatellite sequence length during DNA replication. Deficiencies in the activity of the MLH1 or MSH2 MMR proteins are the most common cause of MSI-High tumors, although deficiencies in PMS2 and MSH6 activity have also been observed. EMAST, on the other hand, can also be found in MSI-low and microsatellite stable tumors that do not demonstrate MMR defects due to these proteins. Therefore, there is incomplete overlap between EMAST and MSI and the MMR proteins responsible for the two instabilities differ.

EMAST has been associated with inflammation^3–6,8^ and EMAST^+^ tumors have also been shown to have a higher CD8^+^ T cell infiltrate than EMAST^−^ tumors^6^. The presence of EMAST is associated with MSH3 dysfunction^3,11^, as MSH3 is involved in the repair of insertion/deletion loops of 2–16 nucleotides (for review see^1^). Nuclear heterogeneity of MSH3^6^ and/or loss of MSH3 expression^4^, as well as re-localization of MSH3 to the cytoplasm from the nucleus in response to oxidative stress^12^ and/or inflammatory cytokines^13^, have all been correlated with the presence of EMAST. In addition, heterogeneity in tumor cell expression of MSH3 positively correlates with the number of tetranucleotide satellite markers mutated in the tumor^5^. There is also some evidence that EMAST might be relevant to the racial disparity observed in the incidence and severity of CRC. African Americans with rectal cancer are more likely to have EMAST^+^ lesions when compared to their Caucasian counterparts^8^. Although CRC is a potentially preventable and curable disease, its occurrence and mortality prove that it remains a major health issue^14^.

One of the principle limitations of studying EMAST is the difficulty in obtaining adequate numbers of human patient samples, particularly from under-represented groups, and the lack of any currently available animal model of cancer that is known to display EMAST. The presence of EMAST in an animal cancer model would significantly enhance current ability to analyze the underlying molecular mechanisms and inflammatory responses associated with the EMAST biomarker. In this study, we describe the design and assessment of a method for identifying EMAST in animal models of cancer, and show for the very first time that EMAST can be studied in an animal model of CRC.

## Results

To identify characteristics of human EMAST sequences that could be used to identify potential EMAST repeats in other species, a thorough review of the literature regarding EMAST was done. This revealed that the most common cancer type studied is CRC and the criteria for calling a tumor EMAST^+^ varies from one to two unstable repeat sequences in a panel of at least five repeat sequences analyzed^2,4–9,11–13,15–26^. The most commonly used tetranucleotide repeat was AAAG and the repeat length varied from 7 to 29. When ENSEMBL was used to evaluate the sequences used in human studies, it revealed that most of the sequences had evidence of previous instability (Fig. S1). This instability presented itself primarily in the form of single nucleotide polymorphisms (SNPs), but insertions/deletions of repeats (indels) and copy number variations (CNV) were also observed in some sequences. The sequence of the repeats, while often reported as AAAG, AGAT, etc., in the literature, is reported in ENSEMBL on the opposite strand. Thus, these repeats are referred to as CTTT, ATCA, etc., throughout this study.

**Figure 1:**
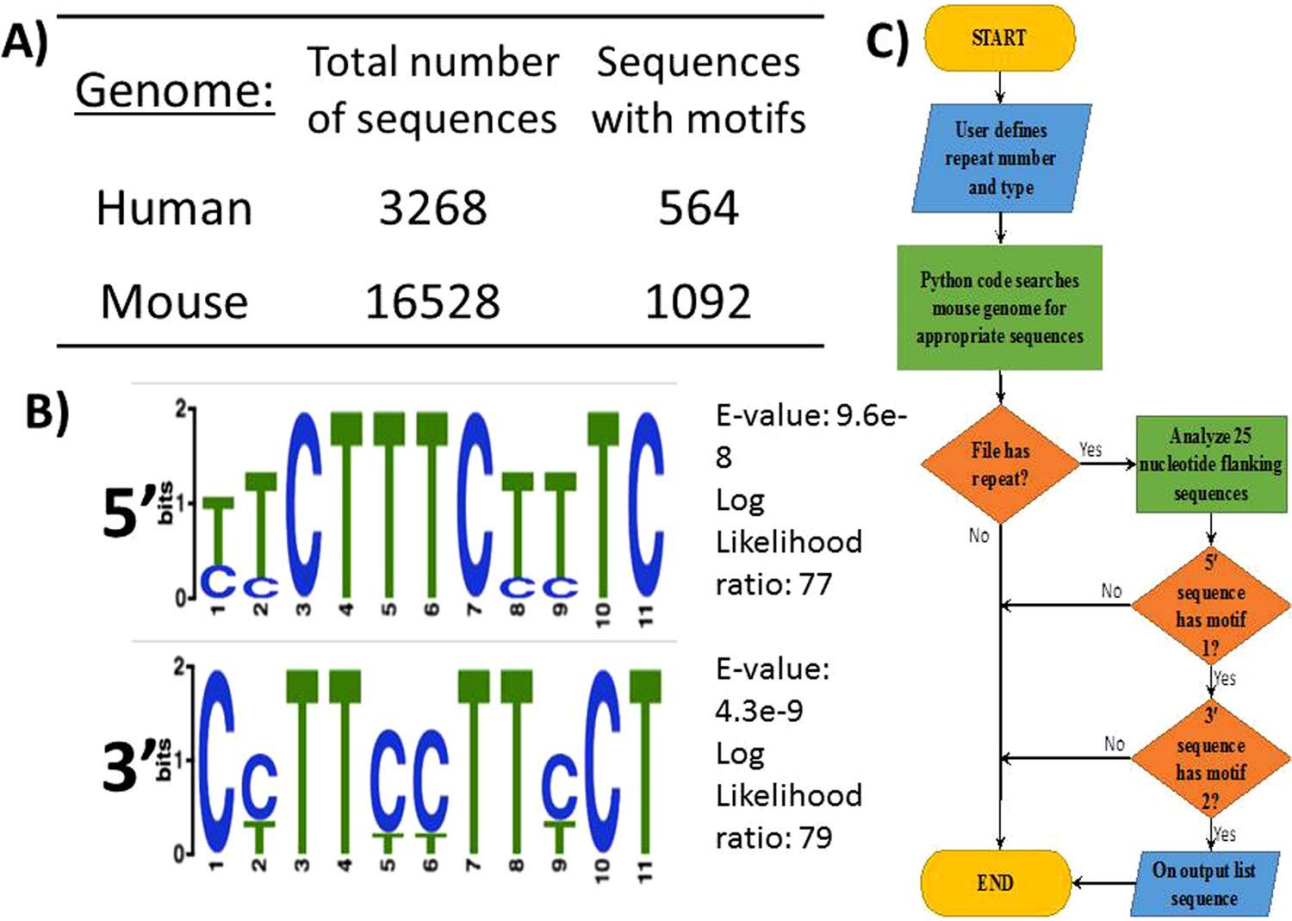
Developing an algorithm to identify potential EMAST CTTT repeats in animal genomes. Novel motifs in flanking regions provided an additional parameter, beyond repeat number and type, to identify unstable tetranucleotide repeat sequences in the mouse genome. (**A**) The number of CTTT repeat sequences found in human and mouse genomes, with and without the identified motifs. (**B**) Sequence logo from Meme showing the 11 nucleotide motifs found in 5′ and 3′ flanking regions of known human CTTT EMAST loci (E-values < 0.001). (**C**) Flow diagram of the programming logic used once motifs were added, from data input to output. The algorithm follows a basic search of the input animal genome for repeat sequences that match the user’s input in type and length. The queries that match are then analyzed for both flanking motifs. If both motifs are found, the algorithm calculates the percentage similarity between the found animal sequence to known human sequences before listing the sequence and values as output. It is important to note that the algorithm as written only reads one strand of DNA; it can be easily modified to find CTTT repeats on the other strand as well.

To determine the key characteristics of these EMAST sequences, 15 of the most commonly used repeats shown to be unstable in tumors were analyzed (Fig. S1). Out of 15 sequences, 7 were CTTT (*i.e*. AAAG) repeats. 10 out of 15 showed previous evidence of instability in the form of SNPs and all of them were ≥10 repeats in length. 4 out of 15 sequences were ATCT (*i.e*. AGAT) repeats, the second most common type observed. Most of these EMAST repeat sequences were found in non-coding regions, and it was previously shown that those found in coding regions most often do not display EMAST^26^. A comparison of both genomes verified that CTTT tetranucleotide repeat sequences also exist in non-coding regions of the mouse genome (Fig. 1A). The abundance of sequences with repeat length between 10 and 25 for each chromosome in both genomes was analyzed to understand the lower and upper limit of CTTT tetranucleotide repeat lengths for both species and to determine whether they are similar (Fig. S2). This revealed that the mouse genome contains more CTTT repeats between 10 and 25 in length than the human genome but tetranucleotide repeats of longer length (*i.e*. >18 repeats) were less abundant than shorter repeats in both species.

Flanking regions of repeat sequences that can become unstable have been shown to be unique compared to other similar, but stable, repeat sequences^27–29^. The specific property in the 25 flanking nucleotides that leads to instability is unknown, but it is thought that these sequences influence the function of the DNA MMR protein, MSH3^28^. Because of these observations, the flanking regions of the commonly used EMAST sequences were analyzed in order to discover a novel parameter that could be incorporated into the algorithm using Meme^30^. Analysis of the 5′ and 3′ CTTT flanking sequences revealed that they were repetitive, C/T rich, and contained novel motifs (Fig. 1B). The 5′ motif chosen was an 11 nucleotide sequence found in 6 out of 7 flanking sequences and had the lowest Meme-calculated E-value of 9.6 × 10^−8^ of all motifs generated. The similarly chosen 3′ motif was also 11 nucleotides in length and found in 6 out of 7 sequences, with an E-value of 4.3 × 10^−9^. It is important to note that no significant motifs were identified using the human ATCT EMAST repeats, perhaps because there are only four known and this number is not great enough to identify common elements in the flanking regions.

In order to find potential EMAST sequences in the mouse genome, a search algorithm was designed in Python (see Methods) that incorporated the characteristics of EMAST loci, including repeat length (*i.e*. ≥10), type (*i.e*. CTTT), and the 5′ and 3′ sequence motifs (Fig. 1C). The algorithm was run with and without the inclusion of the motif information. Inclusion of motif information significantly reduced the number of potential EMAST sequences found by the program, by 78% and 91% in the human and mouse genomes, respectively, from the total repeat sequences identified when the algorithm did not include the motifs (Fig. 1A).

To determine the accuracy of the algorithm, the Python script was run with the unmasked human genome (where repetitive DNA nucleotides are not changed to N for purposes of comparison) to confirm whether the known human EMAST loci could be found. 728 human sequences, of which 100 have a position weight matrix score within the top tenth percentile, were found^31^, including all seven known AAAG repeat human loci (S3). When the program was run with the unmasked mouse genome, the highest match on similarity to known human sequences was ~99%. Sequences from each chromosome that were ≥85% similar and at least 13 repeats long were selected for further analysis. Previous studies had revealed that the longer the length of the repeat, the more likely it was to be unstable^29,32^. Of the 23 longest mouse sequences analyzed, 19 showed previous evidence of instability in ENSEMBL, in the form of indels, SNPs and CNVs (S4). In order to investigate these sequences *in vivo*, a panel of seven CTTT repeats that had previous evidence of instability in the form of either CNV or specific CTTT tetranucleotide indels (S5) were chosen for further study. Two control, randomly chosen, ATCT, repeats (Ch18R14.1 and Ch18R14.2) not identified by the algorithm were also analyzed.

**Figure 2:**
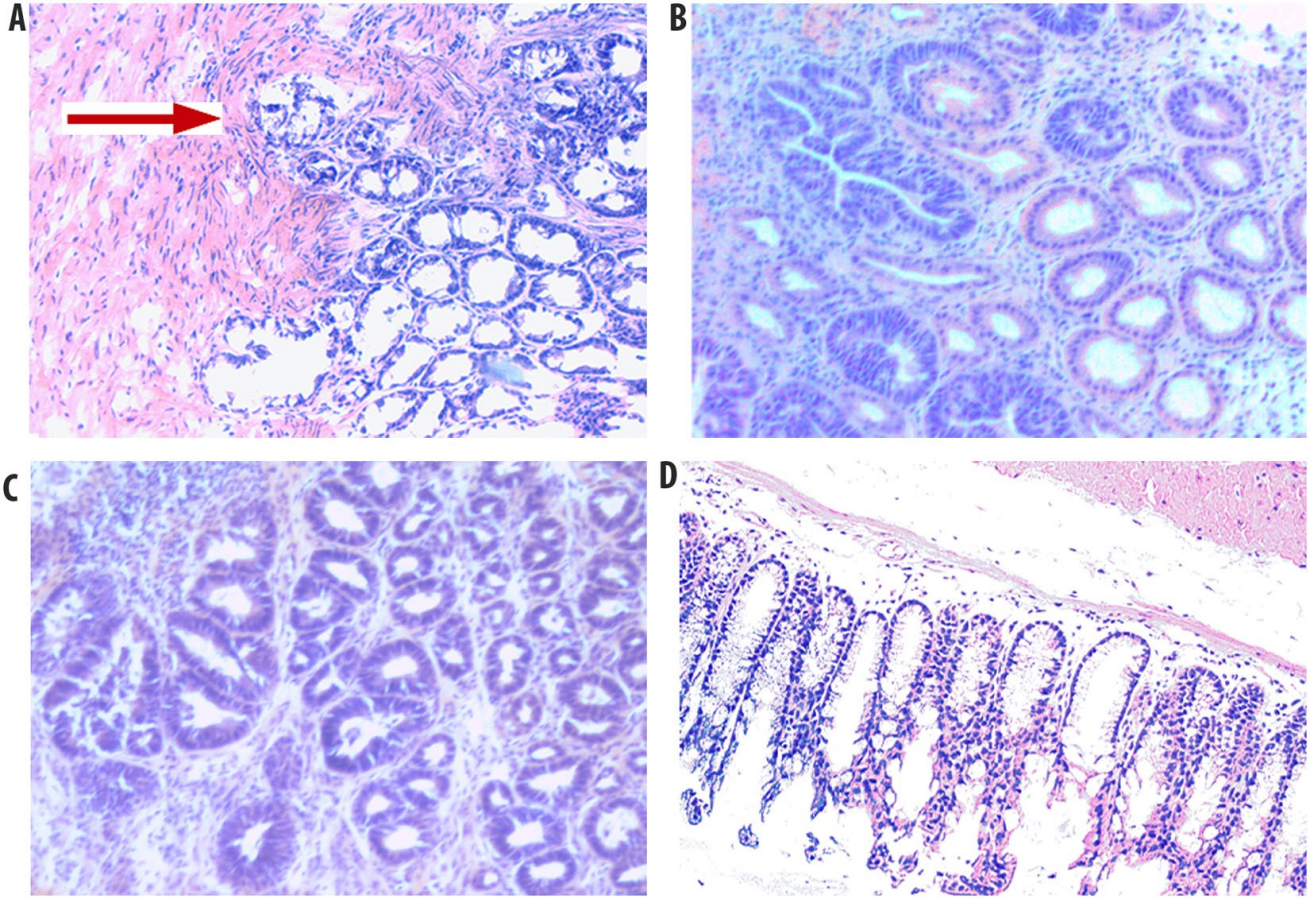
Histology of tumor sections from mouse colon. 5 µ sections were stained with Meyer’s hematoxylin for histological evaluation. Representative samples shown: (**A**) Invasive tumor tissue sample T3 from mouse #T2. The red arrow indicates where the tumor has invaded into the submucosa; (**B**) non invasive tumor tissue sample T2 from mouse #122; (**C**) non invasive tumor tissue sample T7 from mouse #134; (**D**) normal mouse colon tissue sample N2 from mouse #122. All pictures were taken at 100x magnification.

**Figure 3:**
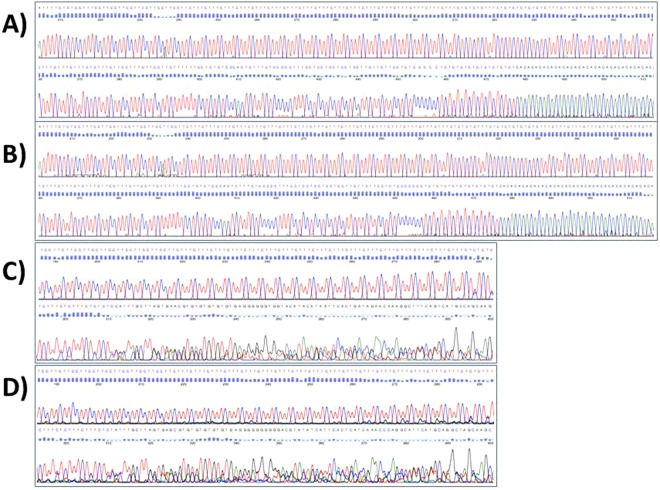
Sequencing chromatographs of marker C9R17 and C10R18. (**A**) Chromatograph showing sequencing peaks of normal mouse tissue sample N6 with marker C9R17. (**B**) Chromatograph showing sequencing peaks of tumor mouse tissue sample T6 with marker C9R17. (**C**) Chromatograph showing sequencing peaks of normal mouse tissue sample N5 with marker C10R18. (**D**) Chromatograph showing sequencing peaks of tumor mouse tissue sample T5 with marker C10R18.

The mouse model of CRC used for further analysis of these repeat sequences was the TS4Cre × Apc^lox468^ mouse model of CRC^33–35^. This mouse model utilizes colon-specific expression of the CRE recombinase to delete exons 11–12 from one copy of the *Apc* gene, resulting in a frame-shift truncation of the product, similar to what occurs in human cancer. Colons were harvested from seven mice and macroscopically normal, but tumor adjacent, and tumor tissues were isolated (S6). Larger tumors (≥5 mm in size) were selected for analysis, using the rationale that EMAST has been observed in larger, more advanced lesions in patients and, therefore, it would be more likely to find EMAST in larger lesions from the mouse model. Three of the seven tumors chosen were shown to be invasive on histological examination, demonstrating they were adenocarcinoma, while the other four tumors were large but not invasive (Fig. 2). Initial PCR and sequencing results using normal mouse colon DNA confirmed that these seven repeats could be studied in the mouse tissues, despite the repetitive nature of these potential EMAST sequences. Sequencing assays for each marker in the panel were done in each tissue sample, and representative sequencing data for markers C9R17 and C10R18 are shown in Fig. 3. Some sequencing chromatographs showed clean peaks throughout the repeat region and beyond in both normal and tumor samples (Fig. 3A,B), whereas others showed heterogeneous peaks towards the end of the repeat region and beyond in both normal and tumor samples (Fig. 3C,D). Those repeats that showed instability in normal and/or tumor DNA, i.e. those with repeat variations observed, were more likely to show heterogeneous peaks in either or both normal and tumor tissue. This observation was expected, as mice displaying instability do not necessarily have the same repeat number for a particular marker on both autosomes, nor do all cells from a tissue undergo the same insertions/deletions. Together, these data suggested that at least some of the algorithm-identified repeats were indeed unstable, and potentially susceptible to EMAST.

**Figure 4:**
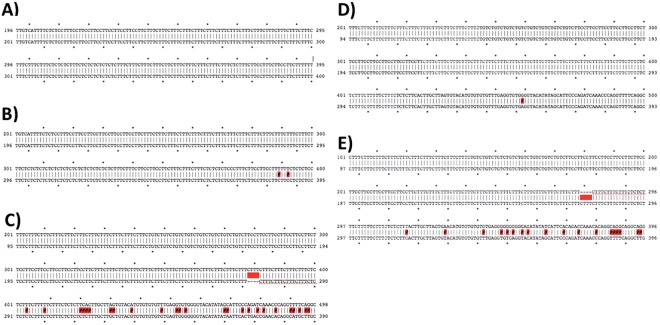
Sequencing alignments of marker C9R17 and C10R18. (**A**) Sequence alignment of marker C9R17 published sequence (top) to sample N6 (bottom). (**B**) Sequence alignment of marker C9R17 published sequence (top) to sample T6 (bottom). (**C**) Sequence alignment of marker C10R18 published sequence (top) to sample N1 (bottom). (**D**) Sequence alignment of marker C10R18 published sequence (top) to sample N2 (bottom). (**E**) Sequence alignment of marker C10R18 sample N1 (top) to sample T1 (bottom).

To detect EMAST, comparisons of macroscopically normal and tumor tissue from the exact same mouse were made, as is done in human studies. Alignments of some normal and tumor repeat sequences to the published genomic sequence (*i.e*. the sequence obtained from NCBI for the C57Bl/6 mouse genome) were clean, without major mismatches or gaps in the repeat or flanking regions (Fig. 4A,B). However, some normal samples displayed tetranucleotide gaps indicating a difference in the repeat length when compared to the published sequence (Fig. 4C), while others did not (Fig. 4D), indicating that the repeat length could be variable in the macroscopically normal colon tissue of individual TS4Cre × Apc^lox468^ mice. Therefore, only alignments between normal and tumor sequences from matched samples that uncovered gaps of four, or multiples of four, nucleotides were used to indicate the occurrence of EMAST (Fig. 4E). EMAST was determined in this manner because in human studies, tumors are considered EMAST^+^ when the repeat number varies from surrounding normal tissue DNA. All differences found between each pair of normal and tumor DNA, or normal DNA and the published sequence, were confirmed in independent PCR and sequencing reactions. Table 1 provides a summary of all sequencing data in terms of the number of tetranucleotide repeats found for the seven markers in each sample assayed. Five out of seven markers identified by the algorithm and tested showed instability in macroscopically normal colon tissue from the individual TS4Cre × Apc^lox468^ mice. Five out of seven markers also displayed EMAST in the seven tumor samples assayed, based on comparison with normal tissue, although only four markers that showed EMAST overlapped with those that showed instability in individual mice. Five out of seven tumors assayed were determined to be EMAST^+^ with at least one marker out of seven unstable. It is very important to note that two randomly chosen ATCT repeats, not identified by the algorithm, showed no evidence of instability, suggesting that the algorithm does identify repeat sequences that have the potential to demonstrate instability.

**Table 1:** The number of TCCC repeat lengths found in the seven loci tested from each mouse normal and tumor colon tissue sample assayed.

Loci Name^a^	Published # of Repeats^b^	Sample Type	Samples						
			1	2	3	4	5	6	7
C6R16	16	Normal	15*	16	15*	15*	15*	16	15*
		Tumor	15	**15**	15	**16**	15	**15**	15
C8R17	17	Normal	17	17	17	17	17	17	17
		Tumor	17	17	17	17	**18**	17	**16**
C9R17	17	Normal	17	17	17	16*	17	17	17
		Tumor	17	17	17	16	17	17	17
C10R18	18	Normal	17*	18	17*	17*	17*	17*	18
		Tumor	**18**	**17**	17	17	17	17	18
C14R15	15	Normal	15	15	15	15	15	16*	15
		Tumor	15	15	15	15	15	**15**	15
C14R16	16	Normal	15*	16	15*	16	16	15*	15*
		Tumor	**16**	**15**	15	16	**15**	**14**	**16**
C19R16	16	Normal	16	16	16	16		16	16
		Tumor	16	16	16	16	16	16	16
Ch18R14.1	14	Normal	14	14	14	14	14	14	14
		Tumor	14	14	14	14	14	14	14
Ch18R14.2	14	Normal	14	14	14	14	14	14	14
		Tumor	14	14	14	14	14	14	14
C10R18	18	Parental	18	18	18	18	18	18	18

^a^Loci name (generated from the chromosome #Followed by the repeat length), the published number of repeats, the sample type, and sample #1–7, as reported.

^b^Published number of repeats for comparison. For each locus, the number of repeats in each normal sample can be compared to the published genomic data, and to the corresponding tumor data (indicated in the row directly below) to determine sequence instability and/or EMAST.

^*^Results determining instability between individual mice in normal samples are marked with an asterisk.

Bold – Results identifying EMAST, as determined by human studies (comparison of normal DNA sequence *vs*. tumor samples).

To investigate the unexplained observation that macroscopically normal colon tissue from individual TS4Cre × Apc^lox468^ mice displayed instability further, one marker, C10R18, was investigated in seven colon DNA samples obtained from normal parental APC^lox468^ mice. Parental animals do not have any defect in Apc expression and, thus, don’t develop colonic polyps. None of the parental animals displayed any instability in the C10R18 marker (Table 1), indicating that the algorithm did not identify repeats that were inherently unstable in normal mice, but rather ones that display instability in diseased colons only. Overall, the results presented here demonstrated that the algorithm developed successfully identifies EMAST repeats in animal genomes and confirm, for the very first time, that EMAST can be studied in an animal model of cancer.

## Discussion

In these studies, an algorithm has been developed that identified EMAST repeats in the mouse genome. Use of the repeat type, repeat length, and the inclusion of identified sequence motifs in 5′ and 3′ flanking sequences successfully identified CTTT (*i.e*. AAAG) tetranucleotide repeats that display instability. In fact, 86% of sequences studied further (*i.e*. 6/7) displayed instability in the TS4Cre × Apc^lox468^ mouse model of colon cancer while neither of two randomly selected controls displayed instability. These data demonstrate that use of the motifs selects the animal CTTT tetranucleotide repeats that are more similar to known human EMAST sequences. It can also be noted that this refinement was not biased towards repeat length, as the inclusion of the motifs did not prompt the algorithm to select longer repeat sequences despite the repetitiveness of the motifs. The program selected sequences of similar length in both human and mouse genomes.

Study of seven repeat sequences in the colon tissue from the TS4Cre × Apc^lox468^ mouse model of CRC verified that five of those studied display EMAST as defined in human patient studies. That is, five of the markers had a variable number of repeats between normal and tumor tissue from the same mouse. However, the observation of instability in macroscopically normal tissue was unexpected. Because no instability in the C10R18 marker was observed in parental Apc^lox468^ mice that lacked colonic lesions, this observation strongly suggests that the instability of the repeats observed in macroscopically normal colon tissue of TS4Cre × Apc^lox468^ mice was more likely due to a diseased, possibly inflamed^36^, colonic environment in the mouse model and not an inherent property of the sequences to destabilize in normal mouse colon tissue. In retrospect, this observation might could have been predicted, as microsatellite instability, including EMAST, has been observed in dysplastic but non-malignant tissues, such as those in ulcerative colitis, that are associated with inflammatory and/or oxidative stress (for review see^3^). These conditions are known to lead to cancer and to promote aggressive disease. This does suggest, however, that in animal models of cancer, where multiple lesions are possible in an organ due to the widespread nature of the genetic defect, it might be best to test for ‘normal’ repeat numbers using a non-affected tissue. DNA isolated from tail tissue or peripheral blood could be more appropriate than colon tissue in this specific mouse model of cancer. Although it was not tested, this hypothesis might indicate C9R17, which demonstrates variability in the normal tissue from one mouse, may very well be an additional EMAST marker in mice. While none of the seven tumors tested displayed EMAST with this marker as defined by human studies, one tumor, T4, did display a variable number of repeats when compared to the published sequence.

A limitation of this study is the lack of ATCT (*i.e*. AGAT) tetranucleotide repeats in this new panel of mouse EMAST repeats. The reason for this was that statistically significant novel motifs could not be identified from the flanking regions of the four human AGAT sequences that have been used previously. As a result, the program was unable to provide useful AGAT repeat sequences that have the potential to undergo instability. This is further highlighted by the observation that two randomly chosen AGAT repeats that met the requirement of repeat length did not display EMAST in our mouse model. Future studies will need to address this, as well as determine the incidence of EMAST in mouse models of CRC to determine if occurrence is similar to that seen in humans. Analyses into the tumors themselves need to be done to determine if similar characteristics of inflammation and MSH3 dysfunction can be seen in mouse EMAST^+^ tumors and to further our understanding of the underlying mechanisms of EMAST. Due to the versatility of the algorithm designed, it is also now possible to build panels of EMAST markers in animal models other than mice. In addition, the algorithm may identify better EMAST markers for use in human cancer patients, perhaps facilitating the formation of a consensus panel of markers which is needed to standardize studies in this field^1^. It is also important to note that trinucleotide repeats can also show EMAST. The identification of unstable trinucleotide repeats, which are important in neurological and neuromuscular diseases (reviewed in^37^) in addition to being EMAST markers in cancer, might also benefit from the approach developed here.

In conclusion, a streamlined approach to discovering potential species-specific EMAST sequences that can be used to develop panels of EMAST markers in any animal model of cancer has been developed. In addition, the results shown here develop a panel of EMAST markers for use in mouse models of cancer and show, for the very first time, EMAST can occur in a mouse model of CRC.

## Materials and Methods

### Mouse model

TS4Cre x Apc^lox468^ mice were bred using TS4Cre homozygous mice and Apc^lox468^ (called cApc hereafter) homozygous mice. cApc homozygous mice have been modified to have the 11^th^ and 12^th^ exons of the Apc gene floxed^30^. TS4Cre homozygous mice express Cre recombinase specifically in the intestinal and colonic epithelium under a fatty acid binding protein gene promoter^31^. Cross-bred mice will undergo Cre-mediated recombination and produce one Apc gene that is truncated at codon 468. These mice develop polyps confined to the distal colon/rectum and distal ileum^32^. All animal experiments and breeding were performed in accordance with relevant guidelines and regulations and approved by the San Diego State University Institutional Animal Care and Use Committee.

Mice were sacrificed at 5 to 10 months of age. Colons were harvested and tumors were documented by size in mm (diameter) and isolated. Seven large tumors (>5 mm) were selected for this study; three of the selected tumors (T1, T3, and T6) were shown to be invasive adenocarcinoma while the remaining four were not invasive and were, thus, large adenomas. Normal tissue was isolated from macroscopically normal, tumor-adjacent, colon tissue in the distal colon.

### DNA extraction

Tumors and adjacent normal colon tissues were used to extract DNA. DNA extraction was done using the REDExtract-N-Amp Tissue PCR kit (Sigma-Aldrich #R4775, Missouri, USA) using small sections of tumor and normal tissue. First, 50 µl of Extraction buffer and 12.5 µl of Tissue Preparation Buffer are added to a PCR tube and mixed. The tissue to be digested was then submerged in the solution. The tube was then put in the PCR machine for the following incubations: 10 minutes at 37 °C, 3 minutes at 95 °C and then cooling to 55 °C. Once completed, 50 µl of Neutralization buffer was added to finish the reaction. All reagents were provided in the kit.

### Collecting parameters for EMAST sequences

Review of previously published EMAST papers in various cancers (see manuscript text) provided a list of known human EMAST sequences which were analyzed using NCBI to get further information regarding tetranucleotide repeat type and length. Previous evidence of known instability or overlap with known genes/novel RNAs in these sequences were found using ENSEMBL (http://uswest.ensembl.org/index.html). Previous evidence of instability is derived from published sequence variations from population studies found via sequence search in ENSEMBL.

### MOTIF analysis

5′ and 3′ flanking sequences of known human EMAST sequences were analyzed using the MEME Suite 4.11.1 program for motif discovery (http://meme-suite.org/tools/meme). FASTA sequences for flanking sequences were collated and run using the normal discovery mode. Analyses were done in categories of tetranucleotide repeat type.

### Python programing

Python code written using Python V 2.7.9. Code (http://www.python.org) was written to open and read FASTA files, and iterate through user provided files to search for user defined tetranucleotide type & repeat number. Appropriate sequences were then enlarged to include 25 nucleotide flanking sequences on either side. These flanking sequences were then iterated to search for the presence of the appropriate motif. If all properties were met, the sequence was then written to an excel file as output.

### Analysis of sequences

The unmasked mouse genome FASTA files were downloaded from NCBI as chromosomes. These files were put in the same directory as the python program and were used in the program’s analysis of CTTT repeats. Output excel files contained lists of found sequences that matched search criteria. The longest sequences with high percentage similarity to known human loci were examined for previous evidence of instability using ENSEMBL. Seven sequences were chosen for further study as described in the text.

### Verification: primer design and PCR protocol followed by sequencing

Primers for the sequences were designed and ordered using the IDT design tool (http://www.idtdna.com/primerquest/home/index). Due to the repetitive nature of sequences, the PCR sequence product was enlarged to 500–600 bp for easy design of unique primers. These primers were blasted against the mouse genome using ENSEMBL before use to verify that the sequences recognized were correct and unique. PCR of seven tumor and adjacent normal samples were done using an extended PCR protocol as follows: initial denaturation at 95° for 5 min; 35 cycles: 95° for 5 sec, 59° for 30 sec, 68° for 10 min; final extension at 68° for 10 min and hold at 4°. PCR products were run on a 1% agarose TAE gel, for 40 minutes at 110 V and imaged to confirm that single products were present. Samples were then sent to ETON Biosciences for purification and forward- and/or reverse-strand Sanger sequencing reactions. Sequence results were then aligned using ApE software (http://biologylabs.utah.edu/jorgensen/wayned/ape/) to identify gaps, if any, and to confirm the correctness of the sequence.

The data obtained in this study are freely available at: https://figshare.com/projects/A_new_method_for_discovering_EMAST_sequences_in_animal_models_of_cancer/34067.

## Electronic supplementary material

Supplementary Material
