# Supplementary material for: A new method for discovering EMAST sequences in animal models of cancer
Source: Sci Rep. 2018 Sep 13;8:13764. doi: 10.1038/s41598-018-32057-2 (PMC6137214; doi:10.1038/s41598-018-32057-2)
Supplement: Supplementary file 1 — Supplementary Material [file 41598_2018_32057_MOESM1_ESM.pdf]

A new method for discovering EMAST sequences in animal models of cancer

Nitya Bhaskaran, Jennifer Luu, Scott T. Kelley, Mohammad W. Khan,
Priyadarshini Mamindla and Kathleen L. McGuire

Supplementary figures

S1 Commonly used human EMAST loci. Loci name, location (chromosome:nucleotide position), properties*, tetranucleotide repeat type, and repeat number. *Properties were determined using the sequence search function in ENSEMBL and includes the evidence of previous instability based on population studies. SNP = Single Nucleotide Polymorphisms.

S2 CTTT Tetranucleotides in the human and mouse genomes. Number of tetranucleotide repeat sequences (CTTT only) between 10 and 25 in length found on each chromosome of the human and mouse genomes.

S3 The algorithm identifies known human CTTT EMAST sequences. Part of the human genome search output containing all 7 CTTT human loci found by the program. Table contains repeat location (chromosome:nucleotide position), repeat number, motif of the loci compared to, loci match and percentage similarity to that loci**.**

S4 Potential mouse EMAST sequences. 23 shortlisted potential mouse EMAST sequences and their associated properties including repeat type, repeat number, location, % similarity to known human loci and previous evidence of instability.

S5 List of mouse sequences and corresponding primer sets and properties. Loci name, tetranucleotide type, number of repeats, chromosome location, forward primer, reverse primer, amplicon size and melting temperatures.

S6 List of tumor and normal tissue samples used. Seven normal colon tissues were isolated from cross-bred mice = N1-N7. Seven tumors, three invasive, T1, T3, and T6, and four large but non-invasive, T2, T4-T5, and T7, were utilized in this study. ‘Normal’ tissues were collected from macroscopically normal colon tissue adjacent to tumors from each of the same mice as the tumors. N1-N7 correspond to T1-T7, coming from the same mice as the tumors.

Figure S1

Loci Name	Repeat Location	Repeat Type	Repeat #	Properties
UT5320	8:125502545-125502957	CTTT	14	none
L17686	7:121603243-121603621	CTTT	18	SNP
D20S85	20:39423030-39423239	CTTT	14	none
D20S82	20:7181877-7182125	CTTT	18	SNP
D8S321	8:128361138-128361357	CTTT	10	none
D9S242	9:124106862-124107060	CTTT	15	SNP
D11S488	11:123513454-123513699	CTTT	10	SNP
D9S252	9:86417402-86417617	ATCT	10	SNP
D9S747	9:110550433-110550625	ATCT	12	SNP
D5S1502	5:26560498-26560733	ATCT	11	SNP
D9S303	9:82276080-82276334	ATCT	12	SNP
L17835	7:57266070-57266290	GGAA	16	SNP
D2S443	2:70560992-70561225	TTCC	14	SNP
D21S1436	21:19970140-19970390	TTCC	12	none
D2S123	2:51061299-51061509	ACAC	11	none

Figure S2

Human Genome

repeats

Chromosome	10	11	12	13	14	15	16	17	18	19	20	21	22	23	24	25
1	26	26	32	32	35	24	26	25	13	9	4	2	1	2	0	1
2	18	20	26	26	27	19	29	19	9	4	0	2	1	2	1	0
3	10	18	11	15	16	18	15	13	9	7	1	2	1	1	0	0
4	12	14	11	20	23	4	14	8	7	4	4	2	1	0	0	0
5	18	11	15	21	19	10	18	9	12	6	5	1	1	1	0	0
6	14	17	16	23	20	19	13	13	4	1	1	3	0	1	0	0
7	13	17	15	18	29	16	20	8	10	3	1	2	0	1	0	0
8	18	12	20	18	21	19	21	7	7	7	8	2	1	1	1	0
9	4	12	9	12	12	12	9	5	3	2	0	1	1	0	1	0
10	10	10	14	22	16	26	10	9	6	1	3	0	0	0	0	1
11	13	10	9	15	10	8	16	12	4	2	1	0	0	1	0	0
12	12	15	19	22	18	19	9	10	9	1	3	2	0	1	0	0
13	5	3	13	17	10	11	10	4	1	1	1	1	0	0	0	0
14	7	9	14	7	18	12	12	7	2	2	1	0	0	0	1	0
15	9	10	6	11	4	17	4	1	1	2	1	0	0	0	0	0
16	9	10	15	16	18	17	13	9	1	3	3	2	0	0	0	1
17	13	6	12	9	10	12	9	5	2	3	1	0	0	1	0	1
18	5	5	8	7	11	7	5	4	3	2	0	1	1	1	0	0
19	9	9	12	8	7	8	8	6	2	4	2	0	0	1	0	0
20	5	8	13	17	8	13	9	8	5	2	0	1	0	0	0	0
21	8	5	10	3	4	6	3	3	0	1	0	0	0	0	1	0
22	1	2	5	0	3	4	6	3	0	0	0	0	0	0	0	0
	239	249	305	339	339	301	279	188	110	67	40	24	8	14	5	4

Mouse Genome

repeats

Chromosome	10	11	12	13	14	15	16	17	18	19	20	21	22	23	24	25
1	126	122	116	110	104	117	93	74	51	35	25	22	12	3	1	1
2	119	91	100	108	128	100	87	79	42	42	25	11	8	7	4	2
3	85	94	101	113	92	97	75	60	55	27	17	15	11	2	3	1
4	94	83	111	107	78	95	81	79	46	42	26	14	5	6	9	2
5	98	84	101	71	79	94	63	51	41	26	17	10	3	1	1	0
6	81	71	80	85	106	66	86	65	38	30	23	6	3	3	2	2
7	72	94	75	86	70	83	73	38	38	21	13	8	9	2	4	2
8	84	86	80	61	71	58	75	51	45	36	26	13	12	9	1	5
9	57	76	81	83	64	62	65	33	21	26	13	5	4	2	0	1
10	102	68	79	64	70	82	49	35	47	14	21	8	6	2	0	0
11	67	78	5	52	77	57	60	51	24	27	13	10	5	3	4	2
12	80	49	80	81	83	55	48	46	40	23	18	13	6	6	3	3
13	74	78	74	70	79	65	41	41	34	17	14	9	7	4	2	1
14	62	72	66	82	69	49	66	51	37	32	21	12	8	8	8	2
15	57	54	60	62	56	49	42	40	30	24	13	6	4	3	1	0
16	67	56	54	57	52	66	54	49	28	20	13	5	4	2	0	0
17	60	59	83	57	45	42	62	37	23	20	10	2	5	4	4	1
18	56	60	51	51	53	61	47	34	27	16	9	5	3	0	0	0
19	35	46	40	29	42	31	25	18	15	13	7	4	1	2	0	2
	1476	1421	1437	1429	1418	1329	1192	932	682	491	324	178	116	69	47	27

Figure S3

Repeat Location	Repeat #	Motif Comparison	Loci Similarity	% Similarity
7:121603433-121603505	18	L17686	L17686	100
8:128361275-128361315	10	D8S321	D8S321	100
8:125502796-125502852	14	UT5320	UT5320	100
9:124106951-124107011	15	D9S242	D9S242	100
11:123513617-123513645	7	D11S488	D11S488	100
20:39423113-39423169	14	D20S85	D20S85	100
20:7181966-7182038	18	D20S82	D20S82	100

Figure S4

Loci Name	Repeat Type	Repeat #	Repeat Location	Similarity to Loci	% Similarity	Instability?
**C1R15**	CTTT	15	*contig* AC122872, 1:37002469-37003462	UT5320	88.57	Single nucleotide polymorphism in C/T
**C2R13**	CTTT	13	*contig* AL844519.9, 2:148814847-148815845	D11S488	94.32	Single nucleotide polymorphism /deletion of C/T
**C3R17**	CTTT	17	contig AC124099.5, 3:25697355-25697948	D20S82	96.15	none
**C3R18**	CTTT	18	contig AC107689.7, 3:49680990-49681659	D20S85	90.22	Single nucleotide polymorphism in C/T
**C4R14**	CTTT	14	contig AL805906.7, 4:85053797-85054662	D11S488	85.56	Single nucleotide polymorphism C/T/G
**C5R14**	CTTT	14	contig AC138175.10, 5:71424879-71425954	D11S488	97.75	Single nucleotide polymorphism /deletion C/T
**C6R19**	CTTT	16	contig AC132365.4, 6:22385624-22386411	D11S488	95.51	Copy Number Variation
**C7R19**	CTTT	19	contig AC161798.6, 7:26418444-26419091	D11S488	96.67	Single nucleotide polymorphism in C/T
**C8R17**	CTTT	17	contig AC165964.3, 8:127169950-127170729	D9S242	90	deletion CTTTCTTTCTTT/TCTT. CNV
**C9R17.CT010488.9**	CTTT	17	contig CT010488.9, 9:13935884-13936685	D11S488	94.19	deletion TCCCTCCT, insertion TC
**C9R17.AC161272.3**	CTTT	17	contig AC161272.3, 9:28640099-28640426	UT5320	91.26	Copy Number Variation deletion
**C9R17.AC161596.2**	CTTT	17	contig AC161596.2, 9:31539782-31540497	D11S488	98.88	Copy Number Variation deletion
**C9R17.AC160137.2**	CTTT	17	contig Ac160137.2, 9:49103170-49103699	D11S488	98.88	none
**C10R18**	CTTT	18	contig AC122372.4, 10:92525242-92525759	UT5320	91.43	Copy Number Variation; loss of allele
**C11R13**	CTTT	13	contig AL646044.13, 11:7713702-7714235	D11S488	95.51	none
**C12R18**	CTTT	18	contig AC102626.9, 12:14296934-14297455	D11S488	98.89	Copy Number Variation, gain of allele
**C13R15**	CTTT	15	contig AC154441.3, 13:111081587-111082128	UT5320	89.8	Copy Number Variation deletion/loss of allele
**C14R15**	CTTT	15	contig CT025590.5, 14:96504786-96505319	D11S488	95.15	Copy Number Variation deletion
**C15R14**	CTTT	14	contig AC137875.13, 15:22485021-22485556	D11S488	95.56	Single nucleotide polymorphism deletions
**C16R15**	CTTT	15	contig AC140198.4, 16:9344558-9345087	D11S488	95.56	none
**C17R16**	CTTT	16	contig AC090881.9, 17:31711368-31711893	L17686	89.62	deletions CTTTCTTTCTTTCTTT
**C18R17**	CTTT	17	contig AC140316.3, 18:10827952-10828487	L17686	91.51	Copy Number Variation loss
**C19R15**	CTTT	15	contig AC132247.3, 19:10651991-10652512	UT5230	90	CNV deletion

Figure S5

Loci Name	Repeat Type	Repeat #	Repeat Location	Forward Primer	Reverse Primer	Amplicon size	Tm
C6R16	CTTT	16	6:22385624-22386411	CCTCATTGAAGCCTA GATCTCTCT	AGTCTGTCTGAAGG CAGCAA	562	63 & 64
C8R17	CTTT	17	8:127169950-127170729	CCTGGAACTGGAGTT ACAAGATG	GCACACTTACATACA CGAGAGG	374	62 & 62
C9R17	CTTT	17	9:28640099-28640426	CAGATCTACCAACTG CACTCTC	ATAGGGAGGAAAGA CACAATGG	590	62 & 62
C10R18	CTTT	18	10:92525242-92525759	GTTATCGCATCCCTG GTTTAGG	CCCAAATCAACACT GCCTCT	641	63 & 62
C14R15	CTTT	15	14:96504786-96505319	TGGTCCTTCCCTGATA TTCTCT	GTCTGGAACAGTGA CTAGAAAGAC	582	62 & 62
C14R15	CTTT	16	14:96504786-96505319***	TGGTCCTTCCCTGATA TTCTCT	GTCTGGAACAGTGA CTAGAAAGAC	582	62 & 62
C19R16	CTTT	15	19:10651991-10652512	GACAGGATTTCTCCAT GTAGGC	GGAGGCAGGTAGAA CTCTATGA	561	62 & 62
Ch18R14.1	ATCT	14	18:21561780-21561893	TTCATCTCACTGCCA GAAGC	CAGTTTGGGAACAA CAGCTC	575	62 & 61
Ch18R14.2	ATCT	14	18:55246025-55246252	CCACCTATAGGGTTG CAATTCG	CTGTGATCTGTCTGT GTGACTG	581	63 & 62

Figure S6

Sample name	Mouse #	Age (months)	Size of lesion (mm)	Invasion Y/N
**T1**	154	8	9	Y
**T2**	122	8	7	N
**T3**	T2	8	6	Y
**T4**	X	8	8	N
**T5**	119	7	8	N
**T6**	141	5	7	Y
**T7**	134	8	8	N
**N1**	154	8	N/A	N/A
**N2**	122	8	N/A	N/A
**N3**	T2	8	N/A	N/A
**N4**	X	8	N/A	N/A
**N5**	119	7	N/A	N/A
**N6**	141	5	N/A	N/A
**N7**	134	8	N/A	N/A
